# Label-free separation of neuroblastoma patient-derived xenograft (PDX) cells from hematopoietic progenitor cell products by acoustophoresis

**DOI:** 10.1186/s13287-021-02612-2

**Published:** 2021-10-15

**Authors:** Franziska Olm, Lena Panse, Josefina H. Dykes, Daniel Bexell, Thomas Laurell, Stefan Scheding

**Affiliations:** 1 Lund Stem Cell Centre and Division of Molecular Haematology, Department of Laboratory Medicine, Lund University, Klinikgatan 26, BMC B12, 221 84 Lund, Sweden; 2 Department of Biotechnology, Technical University Berlin, Berlin, Germany; 3 Division of Haematology and Transfusion Medicine, Department of Laboratory Medicine, University and Regional Laboratories, Lund, Sweden; 4 Division of Translational Cancer Research, Department of Laboratory Medicine, Lund University Cancer Center, Lund University, Lund, Sweden; 5 Division of Nanobiotechnology and Lab-On-a-Chip, Department of Biomedical Engineering, Lund University, Lund, Sweden; 6 Department of Haematology, Skåne University Hospital, Lund, Sweden

**Keywords:** Acoustophoresis, Neuroblastoma, PDX, Patient-derived xenografts, Peripheral blood progenitor cells, PBPC, CTC enrichment, Label-free separation, Purging, Stem cell transplantation

## Abstract

**Background:**

Graft-contaminating tumor cells correlate with inferior outcome in high-risk neuroblastoma patients undergoing hematopoietic stem cell transplantation and can contribute to relapse. Motivated by the potential therapeutic benefit of tumor cell removal as well as the high prognostic and diagnostic value of isolated circulating tumor cells from stem cell grafts, we established a label-free acoustophoresis-based microfluidic technology for neuroblastoma enrichment and removal from peripheral blood progenitor cell (PBPC) products.

**Methods:**

Neuroblastoma patient-derived xenograft (PDX) cells were spiked into PBPC apheresis samples as a clinically relevant model system. Cells were separated by ultrasound in an acoustophoresis microchip and analyzed for recovery, purity and function using flow cytometry, quantitative real-time PCR and cell culture.

**Results:**

PDX cells and PBPCs showed distinct size distributions, which is an important parameter for efficient acoustic separation. Acoustic cell separation did not affect neuroblastoma cell growth. Acoustophoresis allowed to effectively separate PDX cells from spiked PBPC products. When PBPCs were spiked with 10% neuroblastoma cells, recoveries of up to 98% were achieved for PDX cells while more than 90% of CD34^+^ stem and progenitor cells were retained in the graft. At clinically relevant tumor cell contamination rates (0.1 and 0.01% PDX cells in PBPCs), neuroblastoma cells were depleted by more than 2-log as indicated by RT-PCR analysis of *PHOX2B*, *TH* and *DDC* genes, while > 85% of CD34^+^ cells could be retained in the graft.

**Conclusion:**

These results demonstrate the potential use of label-free acoustophoresis for PBPC processing and its potential to develop label-free, non-contact tumor cell enrichment and purging procedures for future clinical use.

**Supplementary Information:**

The online version contains supplementary material available at 10.1186/s13287-021-02612-2.

## Introduction

Neuroblastoma is an early childhood cancer which causes up to 15% of all cancer-related deaths in children [1, 2]. Survival rates in high-risk neuroblastoma patients are poor despite intensive treatment including chemotherapy combined with autologous hematopoietic stem cell (HSCs) transplantation, surgery, radiotherapy, and anti-GD2-therapy [1, 3]. Relapse is the main cause of death and is caused by residual therapy-refractory neuroblastoma cells. However, also graft-contaminating tumor cells in autologous HSC transplants can contribute to relapse as demonstrated by gene marking studies [2, 4, 5]. Of note, circulating tumor cells (CTCs), which can be detected in the blood of about 70% of high-risk neuroblastoma patients and in 50% of stem cell collections [6–9], carry important diagnostic and prognostic information.

**Fig. 1:**
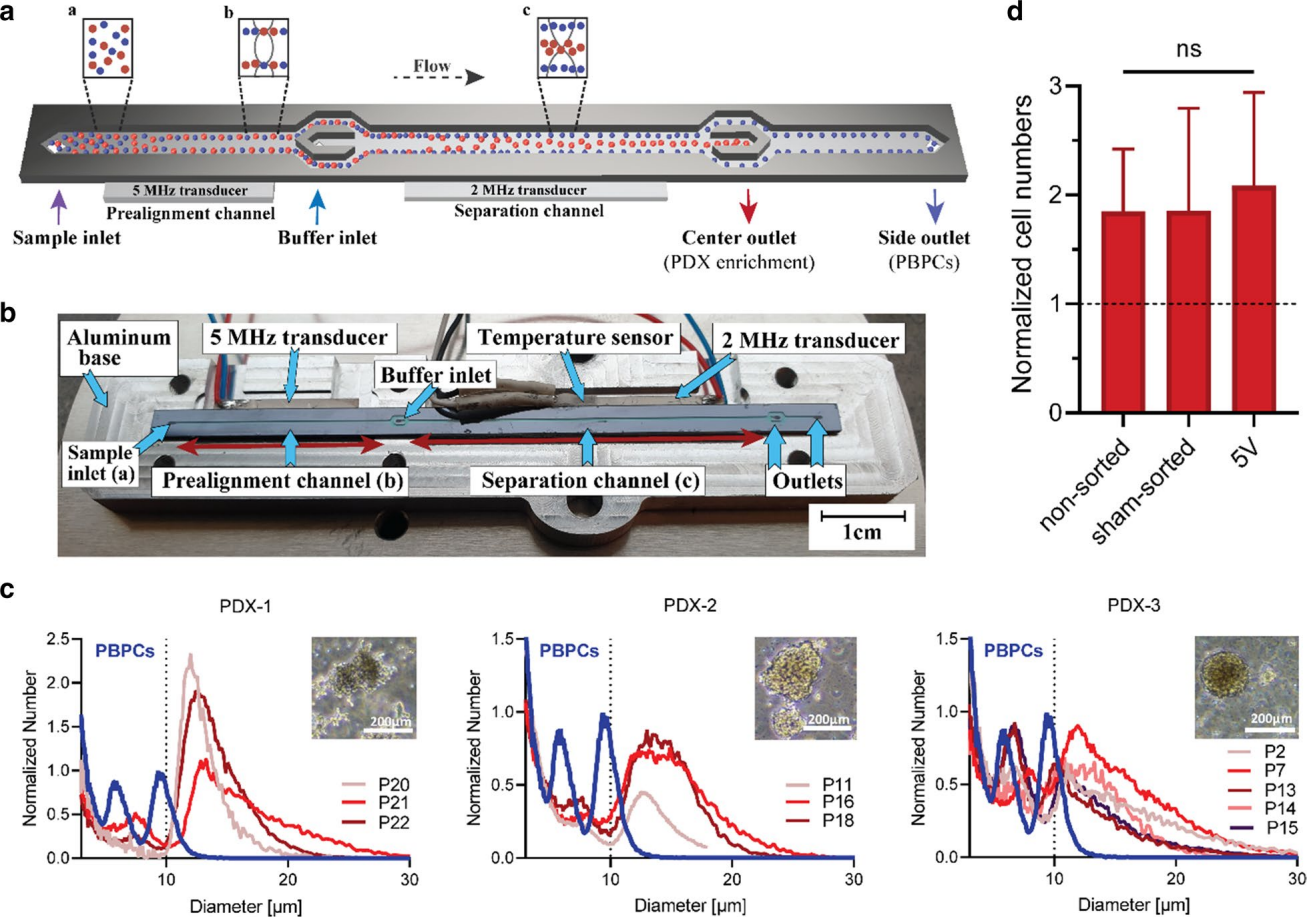
Overview of the acoustophoretic microchip, preserved proliferation capacity and cell size distributions of PDX cells and PBPCs. **A** The schematic illustration shows the acoustophoresis principle. The sample is infused through the inlet (**a**) at 100 µL/min and enters the prealignment channel where the 5 MHz acoustic standing wave induces 2D-alignment of the cells in two lines (**b**). Buffer is centrally infused at 300 µL/min to laterally position the cells close to the side walls, maximizing the lateral migration in the separation channel and hence separation performance. As the cells enter the separation channel (actuated with a 2 MHz transducer), the acoustic force in the standing half-wavelength field induces a movement of the particles towards the channel center (**c**). The effect of the radiation force on the cells is dependent on the cells’ acoustophysical properties, *i.e.*, larger and denser cells (red) move faster in the acoustic field and are directed to the center outlet, while less affected cells are collected in the side outlet (blue, Additional file 1: Equations 1 and 2, Figure S1). **B** Photograph of the silicon-glass chip as used in this study. Visible are the microchip in the open holder, the temperature sensor, transducers, as well as prealignment (**b**), and separation channel (**c**). Arrows indicate inlets (a) and outlet positions. **C** Cell size distributions of PDX cells in different passages (P) and one representative measurement of PBPCs. Cell counts are presented as normalized numbers to the total number of cells acquired per sample. Inserts show representative images of the different PDX cells growing in free 3D spheroid cultures, scale bars represent 200 µm. **D** Analysis of PDX cell proliferation capacity for non-sorted, sham-sorted and acoustically separated PDX-1 cells seeded at identical numbers. Data are normalized to initially seeded cell numbers (dashed line) counted after one week culture (*n* = 3, mean ± SD). Ordinary one-way ANOVA with Kruskal–Wallis multiple comparisons test identified no statistical significances between the different treatments (ns)

We therefore aimed to establish acoustophoresis as a method to effectively enrich neuroblastoma cells from stem cell products for diagnostic and potentially even for therapeutic purposes. Acoustophoresis is a microfluidic technology using standing ultrasound waves in microchannels, which is a gentle, time- and cost-efficient cell separation method. Generally, larger cells and cells with higher density or cells that are less compressible in relation to the surrounding medium will be collected in the center outlet of the acoustophoresis chip, whereas cells that are smaller, cells with lower density or lower compressibility will be collected in the side fractions (Fig. 1A, see also Additional file 1: Equation 1, Figure S1). The extent to which cells with certain acoustic properties will be directed towards the center or will remain closer to the walls of the channel and will be collected in the side outlets, respectively, is determined by the chosen running parameters (*i.e.*, frequencies, voltages).

In contrast to other cell separation methods, acoustophoresis is not depending on cell labelling techniques. Furthermore, acoustophoresis has already been developed towards clinically relevant applications, *e.g.*, separation of platelets from PBPC products, enrichment of circulating tumor cells (CTC) from blood and separation of leukocyte subpopulations [10–13].

Herein, we report advances in label-free acoustic tumor cell separation from PBPC products in a clinically relevant setting, *i.e.,* enrichment of patient-derived neuroblastoma xenograft (PDX) cells for diagnostics and depletion of PDX cells from PBPC grafts, respectively. PDX cells are established from high-risk neuroblastomas and represent a highly valuable neuroblastoma model because cells retain the original molecular and phenotypic characteristics [14–16], in contrast to conventional cancer cell lines that were used in our prior study [17].

Herein, we demonstrate that acoustophoresis of neuroblastoma PDX-containing PBPC products resulted in high tumor cell recoveries and depletion rates at clinically relevant minimal residual disease (MRD) tumor cell contamination frequencies, thus demonstrating the potential of this technology towards clinical PBPC processing.

## Materials and methods

### Neuroblastoma cell cultures

Neuroblastoma PDX-1, PDX-2, and PDX-3 cells were derived from PDX mouse models established from *MYCN*-amplified high-risk patient tumors. The PDX cells (also named LU-NB-1, LU-NB-2, and LU-NB-3) have been previously characterized in detail, and cells were cultured as free-floating 3D tumor organoids as previously described [14, 15]. In brief, cells were cultured in stem cell media [3:1 Dulbecco's Modified Eagle medium and F‐12 Nut Mix GlutaMAX™ supplemented with 2% B27 supplement w/o vitamin A (all Gibco Life Technologies, Carlsbad, CA, USA), 100 IU/mL penicillin, 100 µg/mL streptomycin, and 25 µg/mL amphotericin B (Sigma-Aldrich, St. Louis, MO, USA), 40 ng/mL basic fibroblast growth factor and 20 ng/mL epidermal growth factor (PeproTech EC Ltd, London, UK)]. PDX cells were cultured at 37 °C in a humidified atmosphere in 5% CO_2_.

### Peripheral blood progenitor cells

Leukapheresis samples from healthy donors (*n* = 5, median age 36 years, range: 29–46 years) and patients (myeloma, *n* = 12; Ewing sarcoma; *n* = 1, testis carcinoma, *n* = 1; median age 62 years (range 27–72 years)) were obtained from the Clinical Apheresis Unit, Department of Clinical Immunology and Transfusion Medicine, Lund, Sweden after previously described standard mobilization treatment and PBPC apheresis [10]. All procedures in this study were approved by the Swedish Ethical Review Authority.

### Cell count and size measurements

Cell counts and viability were measured with a NucleoCounter® NC-250™ (ChemoMetec A/S, Allerod, Denmark) according to manufacturer’s instructions. Cell diameters were determined with a Multisizer 3 COULTER COUNTER® (Beckman Coulter, Brea, CA, USA).

### Acoustophoresis chip design and setup

The acoustophoresis and microchip setup is shown in Fig. 1A and was described in detail previously [12, 17, 18]. In this study, a microchip with a 26-mm-long prealignment channel and 43-mm-long main separation channel (long chip) manufactured by Micronit (Enschede, Netherlands) [18] was used for most experiments (Fig. 1B) and compared to a previously used design (short chip) with shorter prealignment and main separation channels (20 and 30 mm lengths, respectively) with otherwise identical dimensions [17].

### Acoustic separation procedure and analysis of sorted cells

The acoustic separation of neuroblastoma cells from CD34^+^/PBPCs was performed as previously described [17]. Optimal actuation frequencies and voltages for the prealignment and separation channel transducers were initially determined in calibration experiments by separation of 5 μm and 7 μm polystyrene microspheres (Sigma-Aldrich). Separation performance was analyzed by flow cytometry (FACS Canto II, BD Biosciences, San Jose, CA, USA). PDX cells and PBPC samples were labelled with directly fluorochrome-conjugated monoclonal antibodies CD45-FITC (clone 2D1), CD34-PE (clone 581), and CD56-APC (clone B159, all BD Bioscience) prior to acoustic sorting. Propidium iodide (Sigma-Aldrich) was used for dead cell exclusion. PBPCs were identified as CD45^+^, and CD34^+^ cells as CD45^low^/CD34^+^ along with scatter properties in accordance with the ISHAGE guidelines [19]. Neuroblastoma PDX cells were identified as CD45^−^/CD56^+^. Data were analyzed using FlowJo v10 software (FlowJo LLC, Ashland, OR, USA). Relative recovery rates were calculated using the Additional file 1: Equations 3.

### Proliferation assay

PDX-1 cell proliferation capacity was evaluated for acoustically sorted, non-sorted, and sham-sorted (ultrasound off) cells by seeding equal cell numbers in triplicates and measuring increase in cell numbers after one week in culture.

### Quantitative real-time PCR (RT-PCR)

RT-PCR was used for sensitive PDX cell detection in apheresis samples with low tumor cell contamination (1:1000 and 1:10,000). Total RNA extraction was performed using the Arcturus™ PicoPure™ RNA Isolation Kit (Applied Biosystems, Waltham, MA, USA), and a NanoDrop® ND-1000 Spectrophotometer (Thermo Fisher Scientific, Waltham, MA, USA) was used to measure RNA concentration and purity. cDNA synthesis was carried out using the SuperScript™ VILO™ cDNA Synthesis Kit (Invitrogen, Waltham, MA, USA) on a T100™ Thermal Cycler (Bio-Rad, Hercules, CA, USA) according to the manufacturer´s instructions. For the RT-PCR, Fast SYBR™ Green Master Mix (Applied Biosystems) was used according to manufacturer's instructions and a CFX96 Touch Real-Time PCR Detection System (Bio-Rad). Primers for *paired-like homeobox 2B* (*PHOX2B*), *tyrosine hydroxylase* (*TH*), *dopadecarboxylase* (*DDC*) and *glyceraldehyde-3-phosphate dehydrogenase* (*GAPDH*) were designed and ordered from TAG Copenhagen A/S (Copenhagen, Denmark), primer sequences are listed in Additional file 1: Table S1. *GAPDH* expression was analyzed simultaneously with NB gene expression on the same samples and served as standardized internal control. Pure PBPCs and PDX cells were included as negative and positive control, respectively, in every experiment. Samples were measured in triplicates, and data are presented as Cq value, which is commonly used for MRD quantification using a standard curve [20].

### Statistical analysis

Results are generally presented as mean ± standard deviation (SD), unless stated differently. Statistical analyses were performed using GraphPad Prism 8 software (Prism version 8.4.0, GraphPad, San Diego CA, US). Statistically significant differences between groups were calculated using one-way analysis of variances (ANOVA) with Kruskal–Wallis or Tukey's multiple comparisons test and are indicated as ns, non-significant, *P < 0.1, **P < 0.01, ***P < 0.001, and ****P < 0.0001.

## Results

### Leukapheresis products and neuroblastoma PDX cells differ in cell size distributions

This study aimed to develop the separation of neuroblastoma PDX cells from “real-life” PBPC products. Cell size is an established and important parameter for efficient acoustic separation since the acoustic radiation force scales with the particle radius to the third power (Additional file 1: Equation 1, Figure S1). Thus, the difference in cell size directly influences the separability of distinct cell types. Therefore, we first measured cell size distributions and identified size differences between tumor and apheresis samples (Fig. 1C). Minor size overlaps between PBPCs and tumor cells were observed for PDX-1 and PDX-2, and a slightly larger overlap for PDX-3 cells. PDX cell size ranged mostly between 10 and 25 µm with an averaged median diameter of 12.5 µm for PDX-1, 13.7 µm for PDX-2, and 11.4 µm for PDX-3 cells. In this analysis, events smaller than 9 µm for PDX-1 as well as PDX-2, and 8.5 µm for PDX-3 were identified as debris and apoptotic or dead cells by flow cytometry and therefore excluded. Cell sizes of the representative PBPC sample ranged between 5 and 12 µm with a median diameter of 6.9 µm (events smaller than 4.5 µm were excluded). PDX cell size distributions were generally constant independent of passage (Fig. 1C).

### Acoustic separation did not affect PDX cell proliferation capacity

Second, we investigated the impact of acoustophoresis on cell function by determining the proliferation capacity of non-sorted, sham-sorted, and acoustically sorted PDX-1 cells (*n* = 3, passages 21, 25, 26). The total increase in cell numbers after one week culture (normalized to numbers of initially seeded cells) was 1.8 ± 0.6, 1.8 ± 1.0, and 2.1 ± 0.9-fold (mean ± SD) for non-sorted, sham-sorted, and acoustically-sorted cells, respectively (Fig. 1D). Differences in proliferation rates were not statistically significant.

### Microchip design and standardized separation settings increase separation efficiency

To progress towards clinically relevant microfluidic separation, an optimized chip design is important to provide an efficient, reproducible, and stable performance. We therefore compared the acoustophoresis chip that was used in our previous neuroblastoma separation study [17] to a new design with a 6 mm longer pre-alignment channel and a 13 mm longer main separation channel. Furthermore, to establish a more standardized separation procedure, we report voltage settings on the main separation channel as % relative to bead calibration setting.

**Fig. 2:**
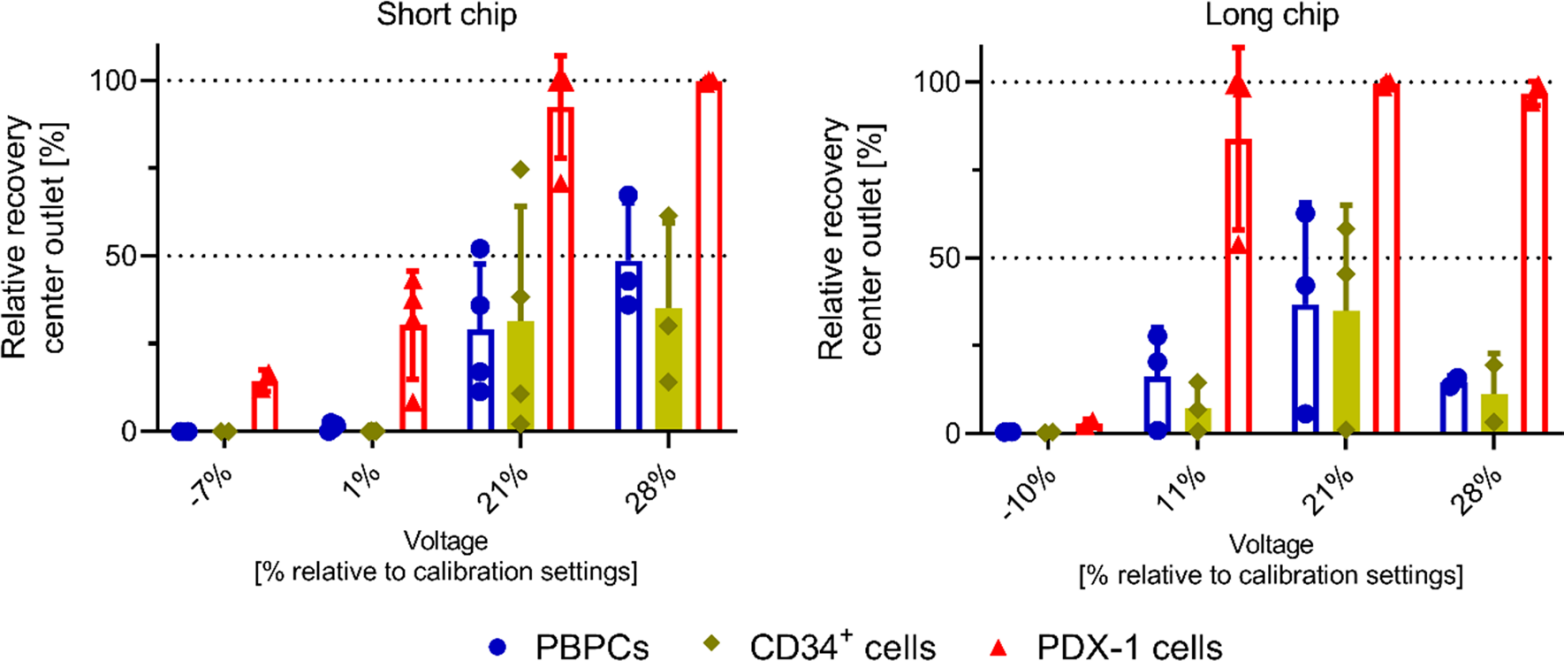
Comparison of the separation performance achieved with the short and longer acoustophoretic chip. Acoustic separation of primary PDX-1 cells spiked into PBPCs at a 1:10 ratio and processing speed of 100,000 cells/min. The relative recovery in the center outlet for PBPCs, CD34^+^ cells and PDX cells for different voltages applied to the separation channel transducer is shown [% relative to calibration settings, ± 3%]. Data are presented as mean ± SD from 3 independent experiments

Experiments were performed at a cell concentration of 1 × 10^6^ cells/mL and with a spiking ratio of 1:10 (PDX-1 cells to PBPCs). When optimizing parameters for high recovery of PDX cells and minimal collection of CD34^+^/PBPCs in the center fraction (“tumor cell outlet”), relative recovery rates of up to 92.5 ± 14.5% for PDX-1 cells were achieved with the short chip (Fig. 2, left, 21% voltage setting), compared to 96.8 ± 3.5% when using the long chip (Fig. 2, right, + 11% voltage setting). Corresponding fractions of PBPC and CD34^+^ cells collected into the center outlet were 29.1 ± 18.6% and 31.5 ± 32.7%, respectively, for the short chip (Fig. 2, left), whereas considerably fewer non-tumor cells were collected into the center “tumor” cell outlet when using the long chip. Here, relative recoveries were only 11.4 ± 11.5% for CD34^+^ cells and 14.7 ± 1.8% for PBPCs (Fig. 2, right). Of note, PBPCs collected in the center “tumor” cell outlet were larger-sized leukocytes, *i.e.,* monocytes and granulocytes, whereas the smaller lymphocytes were successfully recovered in the side outlet. Additionally, separation results were in general more stable with the long chip, which is likely due to the longer time that cells are exposed to the acoustic field in the pre-focusing channel and thus resulted in improved 2D-pre-focusing of the cells before entering the separation channel.

### Standardization allows to optimize cell separation for enrichment of different PDX cells

Based on the chip comparison results, the long chip was chosen for the subsequent experiments to optimize the acoustic separation of neuroblastoma PDX cells from PBPC products. Two main goals were pursued: Firstly, we aimed at the complete removal of residual tumor cells from the graft while minimizing CD34^+^/PBPCs loss from the PBPC product, thus simulating a clinical “purging” scenario. Secondly, we aimed to identify parameters that would allow sufficient tumor cell recovery (enrichment) and purity for potential diagnostic purposes but not necessarily complete tumor cell removal from the graft. As before, initial microbead calibration of the setup served as reference.

**Fig. 3:**
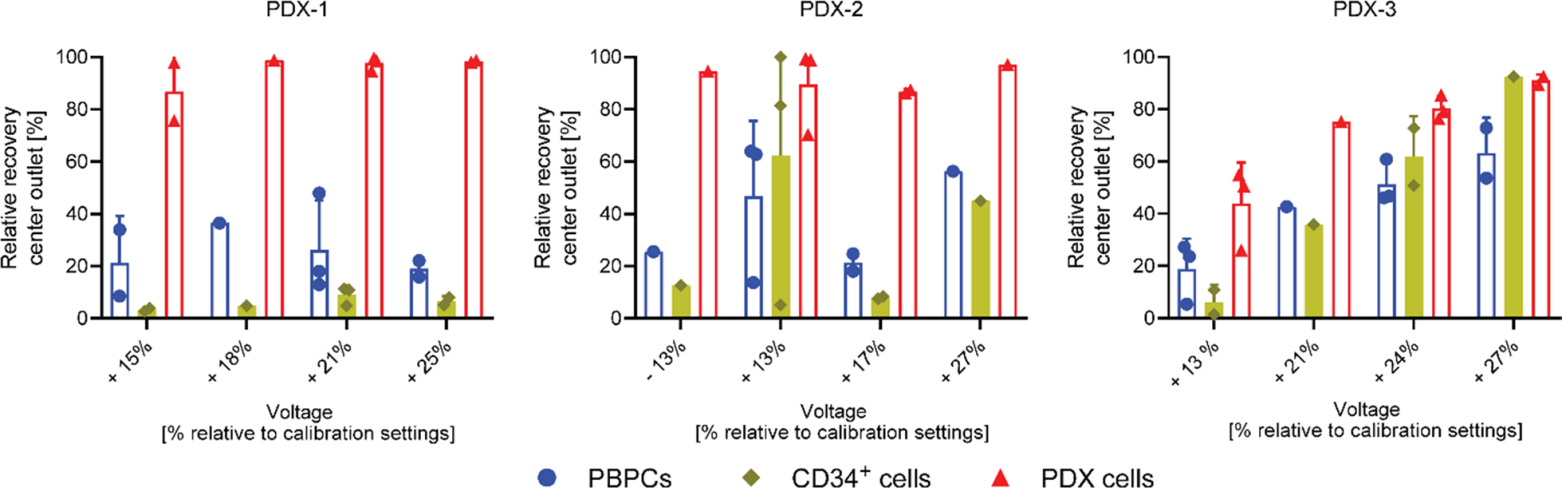
Acoustic separation of primary PDX cells spiked into PBPCs. Relative recovery in the center outlet for PBPCs, CD34^+^ cells and PDX-1, PDX-2, and PDX-3 cells, respectively, with a throughput of 100,000 cells/min for different voltages applied to the separation channel transducer [% relative to calibration settings]. Data are presented as mean ± SD from 3 independent experiments

When PBPC products with 10% contaminating neuroblastoma cells were used, we observed that an increase of 25 ± 1% of the main transducer voltage relative to calibration settings resulted in a relative recovery of 98.4 ± 0.47% of PDX-1 cells in the center (tumor cell) outlet. This corresponded to a 1.8 ± 0.2-log tumor cell depletion of the PBPC product while 93.5 ± 2% of CD34^+^ cells and 81.0 ± 4.4% of PBPCs were retained as indicated by relative center fraction recovery rates of 6.5 ± 2% and 19.0 ± 4.4% for CD34^+^ cells and PBPCs, respectively (*n* = 2, mean ± SD, Fig. 3, left panel). These settings would thus be optimized for purging as well as a diagnostic tumor cell enrichment approach. Nevertheless, high separation performance for PDX-1 cell-contaminated PBPC products was achieved over a wide range of settings.

Promising results, although in a narrower voltage range, were also achieved for PDX-2 cells (Fig. 3, middle panel). When applying a 17.0 ± 1% increased voltage relative to calibration settings, we observed relative recoveries of 86.8 ± 0.9% of the PDX-2 cells, 8.0 ± 0.6% of CD34^+^ cells, and 21.4 ± 4.7% of PBPCs in the center fraction (*n* = 2). This corresponded to a 0.88 ± 0.03-log PDX-2 tumor cell depletion, which is lower when compared with PDX-1 cells, whereas CD34^+^ cell and PBPC recoveries were comparable. The observed high standard deviation for one of the voltage settings (PDX-2 cells, + 13% voltage setting) likely occurred due to unnoticed flow disturbances during the separation process.

PDX-3 cells showed the largest size overlap with PBPCs and, accordingly, separation outcome was inferior as indicated by relative center fraction recoveries of 75.3%, 35.9% and 42.7% for PDX-3 cells, CD34^+^ cells, and PBPCs, respectively (+ 21% voltage setting, Fig. 3, right panel). This corresponded to a maximum tumor cell depletion of 0.6-log, which certainly is insufficient for clinical purging purposes. Nevertheless, PDX-cell enrichment rates would still be sufficient for diagnostic tumor cell analysis with acceptable loss of CD34^+^ cells, especially when considering that only a small fraction of the PBPC product needs to be processed for tumor cell diagnostics.

### Clinically relevant depletion of neuroblastoma cells from PBPCs

Circulating neuroblastoma cells found in stem cell collections of high-risk neuroblastoma patients undergoing autologous PBPC transplantation can be isolated to provide valuable diagnostic information, but also pose a threat of increased relapse risk following transplantation, which emphasizes their depletion from the graft as desirable. The contamination frequency of clinical samples, however, occurs at considerably lower frequencies compared to the 1:10 PDX:PBPC spiking ratio used so far. Therefore, we went on to investigate the efficiency of acoustophoretic removal of neuroblastoma cells from apheresis samples containing PDX cells at clinically relevant MRD concentrations for high-risk neuroblastoma. PDX-1 cells were chosen for these experiments based on their favorable acoustic properties. Experiments with spiking ratios of 1:1000 and 1:10,000 (PDX-1 cells to PBPCs) were performed, subsequently using RT-PCR to quantify tumor cells by measuring expression of *PHOX2B*, *TH,* and *DDC*.

**Fig. 4:**
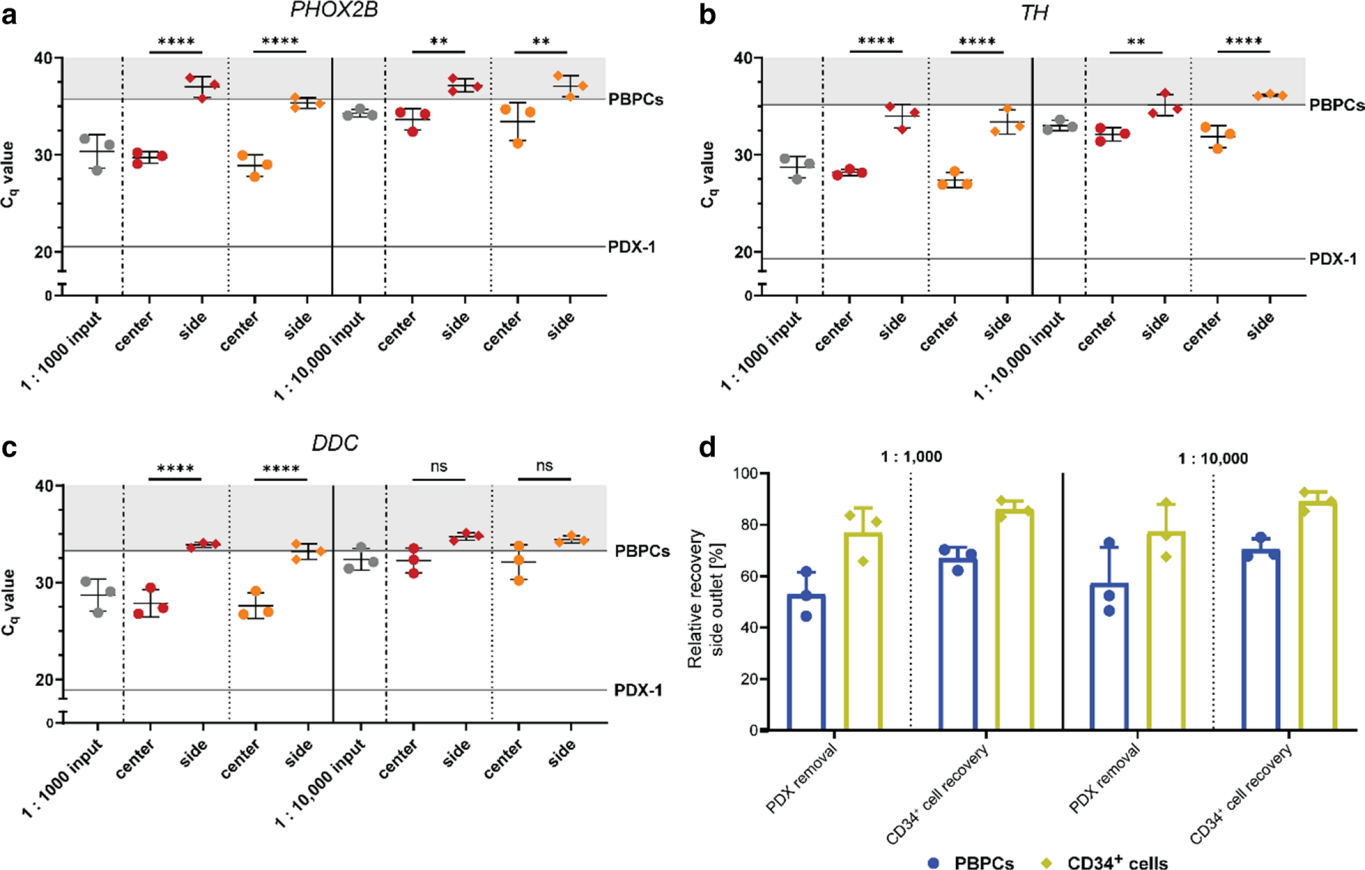
RT-PCR for sensitive detection of PDX cells in PBPCs and respective relative PBPC and CD34^+^ cell recoveries. C_q_ values for *PHOX2B* (**A**), *TH* (**B**) and *DDC* (**C**) of input samples (grey dots), respective center (dots) and side (diamonds) fractions when aiming for complete PDX cell removal from the graft (red symbols) or high CD34^+^ cell recovery (*i.e.*, < 15% loss, orange symbols, *n* = 3, mean ± SD). The detection limit (grey area) is based on respective C_q_ values from non-spiked PBPCs (0% PDX cells, solid line) and Cq values from PDX-1 mark the C_q,max_ (100% PDX cells, solid line). Statistically significant differences are indicated as **P < 0.01 and ****P < 0.0001 (one-way ANOVA with Tukey’s multiple comparisons test), non-significant differences are indicated as ns. (**D**) Relative recovery in side outlets [%] for PBPCs and CD34^+^ cells determined by flow cytometry for RT-PCR experiments (*n* = 3, mean ± SD)

The first approach aimed at maximum PDX cell removal from the graft (collected in the side outlet) into the center outlet (Fig. 4, (A-C) red symbols, (D) PDX removal) for therapeutic approaches, whereas the second approach aimed at high enrichment of sufficiently pure PDX cells in the center fraction for diagnostic purposes while recovering > 85% of CD34^+^ cells in the side fraction (Fig. 4, (A–C) orange symbols, (D) CD34^+^ cell recovery). Relative recoveries [%] in the side outlet for CD34^+^ cells and PBPCs are presented in Fig. 4D. A 2-log depletion of PDX cells from the PBPC sample was achieved, indicated by measurement of expression of all three genes for a spiking ratio of 1:1000 (calibration curve, see Additional file 1: Figure S2). Furthermore, even at lower tumor cell contamination rates (1:10,000 PDX cells to PBPCs) significant depletion of PDX cells and even depletion below background signals, respectively, was reached (Fig. 4A–C, right panels, diamonds).

## Discussion

Circulating neuroblastoma cells can be detected in the blood of about 70% of high-risk neuroblastoma patients and in 50% of stem cell collections [6–9]. The ability to isolate tumor cells from leukapheresis samples of high-risk neuroblastoma patients is not only important to remove viable tumor cells from the graft prior to transplantation but also opens the possibility for diagnostic and prognostic applications of the isolated tumor cells [21–23]. Therefore, efficient, easy to handle and cost-effective isolation methods are desirable.

Acoustophoresis emerged as a gentle, continuous, non-contact and importantly, label-free separation method for cells and particles. Acoustic separation utilizes an acoustic standing wave field generated across a microchannel to direct the movement of suspended cells. The technology has been established towards several clinically relevant applications, *e.g.*, separation of leukocyte subpopulations, isolation of circulating tumor cells (CTC) from blood and PBPCs, as well as separation of platelets from PBPC products [10–13]. Label-free diagnostic isolation of CTCs from solid tumor cells, such as prostate cancer and breast cancer from blood samples has been previously demonstrated in a manner comparable to the CellSearch system and up to clinically relevant scales [11, 12].

Label-free acoustophoretic separability of different cell types primarily depends on differences in the cells’ acoustophysical properties, which are characterized by cell size, density as well as compressibility relative to the surrounding medium (Additional file 1: Equation 1) [24]. We therefore determined the cell size distributions for three different PDX tumor models and identified minor size overlaps for two of the PDXs with PBPCs and a slightly larger overlap with the third PDX cells. Nevertheless, PDX size distributions were comparable to our previously reported data for the neuroblastoma cell line SH-SY5Y [17], and size distributions remained constant independent of passage as well (Fig. 1C). Based on these findings, we expected that PBPCs and PDX cells would sufficiently differ in their acousto-physical properties, thus allowing to be separable by acoustophoresis.

A prerequisite for acoustophoretic processing of clinically relevant samples is that the separation procedure does not affect cell viability or function. We therefore analyzed proliferation capacity of non-sorted, sham-sorted, and acoustically sorted PDX cells. Differences in proliferation were not statistically significant and proliferation rates remained unchanged (Fig. 1D), thus confirming previous data showing that acoustophoresis is a gentle cell separation method that does not affect important cell functions, such as viability or proliferation capacity [10, 17, 25–27].

After confirming cell size differences and preserved cell functions, we then investigated the acoustic separation of the three different PDX cells from PBPCs with the long chip, which showed improved performance compared to the previously used microfluidic chip [17]. The separation results showed that the different PDX models vary in their acoustic mobilities relative to different PBPC donors, but high PDX cell recovery with minimal CD34^+^/PBPC collection (“purging” scenario) was possible for two of the three tested PDX models. The determined size overlaps hence translated into the collection of some larger cells in the PBPC product into the center “tumor” outlet, however, retaining adequate tumor cell purity. As previously reported, PBPC grafts showed varying size distributions between different patients [17]. This, in combination with differences between individual tumors, stresses the need for standardization and optimization of the separation procedure. Herein, using bead separations as a reference point, we identified optimal separation conditions for neuroblastoma PDX cells with voltages between + 17 to + 25% (± 1%) of the calibration settings, which is a good starting point for future tumor cell enrichment studies. Whereas it appears unlikely that effective PBPC purging can be realized for all samples given the current technical standard, development of label-free diagnostic tumor cell enrichment seems to be possible even at this early stage of development of acoustic tumor cell enrichment, which is in accordance with previously published studies [10, 11].

Stem cell collections of high-risk neuroblastoma patients contain considerably lower contamination rates than the 10% spiking rate used herein for sorting optimization [7, 28]. Therefore, we tested if acoustic separation was also efficient for samples with clinically relevant MRD concentrations (1:1000 and 1:10,000 PDX-1 cells to PBPCs). RT-PCR was chosen as analysis method to quantify minute numbers of tumor cells by measuring expression of *PHOX2B*, *TH,* and *DDC* [6]. These genes have been widely used for neuroblastoma MRD detection [2, 6, 29], and it was reported that detectable neuroblastoma associated gene MRD levels in peripheral blood samples were linked to inferior event-free survival [2, 30].

Our results showed that it was possible to enrich PDX-1 cells from PBPC samples spiked at clinically relevant levels, which may be sufficient for diagnostic downstream analysis of stem cell collections from high-risk neuroblastoma patients. At low tumor cell contamination rates, PDX-1 cells were even depleted from PBPC samples to undetectable levels, which indicates that purging could be efficient for some neuroblastoma patients. However, more research will be necessary to better understand the heterogeneity of primary neuroblastoma cells with regard to acoustophysical properties and acoustic separation efficiency. Here, further improvements such as sorting buffer modifications and chip design modifications could provide to the further improvement of the system.

Overall, the data further demonstrate that acoustophoresis as a promising label-free separation method, which enables to separate cells solely based on differences in their acoustophysical properties, could be applied to process PBPC products from neuroblastoma patients [13, 24, 31–33]. The data reported herein are comparable with other reported tumor cell enrichment and purging systems, which are based on different principles such as immunomagnetic beads for either tumor cell removal by selective enrichment or negative depletion by selection of CD34^+^ cells. Using magnetic activated sorting of CD34^+^ PBPCs, which is the most widely used method for PBPC purging in high-risk neuroblastoma, tumor cell depletions rates ranging from 2 log to 4.6 log were reported in different studies for tumor cell lines and clinical samples, respectively [6, 29, 34–36]. A randomized clinical study performed tumor selective purging using immunomagnetic beads with five different monoclonal antibodies to selectively bind neuroblastoma surface antigens in PBPCs from high-risk patients. In pre-clinical modelling of the method, 10–20% tumor cell contaminated bone marrow samples could be depleted by 3–4 logs [6, 37–39]. These depletion rates are somewhat higher compared with the results obtained with our prototype acoustic system reported herein. However, label-based methods are time-consuming and costly, as they require rather expensive reagents and in case of fluorescence activated cell sorting even high-cost advanced machines [40]. Furthermore, although label-based methods provide high sensitivity, a specific surface marker with sufficient surface expression levels needs to be expressed by all tumor cells, which is not always the case considering the high degree of tumor cell heterogeneity and plasticity [6, 41, 42]. On the other hand, other size-based label-free methods that have been tested for diagnostic apheresis including microsieves [43] and inertia-based spiral microfluidics [44] provide for relatively high throughput, yet often at low specificity and adaptability.

The interest in label-free, *e.g*., size-based cell separation methods is also reflected in the application of these technologies in clinical trials for various metastatic cancers, however, not yet applied in the context of neuroblastoma [45]. Membrane filtration, such as the FMSA (flexible micro-spring array, [46]), ScreenCell filters [47] or ISET (Isolation by Size of Tumor Cells, [48]) technologies using varying or defined pore sizes enable to capture heterogeneous CTC populations at low cost. However, there is the risk of clogging, loss of smaller CTCs, high leukocyte contamination, and often decreased cell viability. Here, microfluidic technologies offer the advantage of continues and gentle cell processing, independent of available surface marker expression. Using these techniques allows to isolate heterogeneous mesenchymal as well as epithelial CTC populations label-free, in contrast to immunocapture methods such as the FDA-approved EpCAM-based CellSearch system. Microfluidic technologies are often operated at lower throughput and many devices work with a fixed design with limited options to optimize the system for varying applications. Examples for systems that usually require an adjusted design for each application include inertia- and microstructure-based microfluidic devices, such as the ClearCell FX1 platform (Biolidics, Singapore, [49]) and the Parsortix technology [50]. The limited adjustability usually results in either compromised throughput or high leukocyte contamination of CTCs. In contrast to these passive microfluidic label-free separation technologies, acoustophoresis offers the possibility not only to adjust the flow rates to optimize the separation efficiencies, but also to adjust the acoustic forces in the channel, which allows to fine tune the cut off size of cells collected in the center outlet or the side outlet, respectively. Acoustophoresis also allows to separate cell types based on their differences in cell density, such as red blood cells from white blood cells [18].

This study provides the next important proof-of-principle evidence that acoustophoresis can be developed for clinically relevant cell processing. However, the herein reported throughput of 100 µL/min, equaling 1 × 10^5^ cells/min still needs to be increased and a more automated and standardized operation will be necessary for the technology’s implementation in a clinical setting. In order to realize in-line purging or tumor cell enrichment during a standard leukapheresis procedure, microfluidic throughput will need to be increased by at least 10 times. The separation performance of the long chip presented herein was not yet fully optimized in terms of throughput and could be further explored. To address this limitation and meet these requirements in the future, several of these microchips could be operated in parallel in a more automated setup, allowing for improved ease of operation. It will further be necessary to assess and optimize the standardization procedure using more PDX models or ideally patient material to address the cell heterogeneity seen between patients.

For translation into clinical and routine applications, the acoustophoresis technology needs to be integrated in existing apheresis devices or, alternatively, developed as a stand-alone automated benchtop device for CTC analysis. Efforts to realize the latter are ongoing in Acousort AB (Lund, Sweden), which has developed a number of routine applications in their AcouWash machine platform.

Taken together, we herein report an important next step towards establishing acoustophoresis for clinical neuroblastoma cell separation. We validated the method using PDX cell models from high-risk neuroblastoma patients, showing effective tumor cell selection from PBPC products for two of the three PDX cell types, and we even achieved clinically relevant depletion of tumor cells for contamination rates reflecting the MRD setting.

## Conclusions

This study further emphasizes that acoustophoresis can be developed into an effective label-free separation technology for potential clinical processing of PBPC preparations from high-risk neuroblastoma patients. Acoustic separation does not compromise PDX cell viability or proliferation. The standardized separation procedure allowed not only to deplete PDX cells from leukapheresis samples to undetectable levels, but also to enrich these more real-life tumor cells from the graft for diagnostic purposes. Acoustophoresis provides the potential to be developed into a clinically valuable technology for effective label-free neuroblastoma cell enrichment and depletion from leukapheresis grafts.

## Supplementary Information

**Additional file 1.** **Supplementary Equations. Supplementary Equation 1.** In an ultrasonic standing wave field, the acoustic radiation force F_z_^rad^ acts on a particle that has a non-zero acoustic contrast factor,* Φ*. The magnitude of the force acting on the particle depends on the radius ɑ, κ_0_, ρ_0_, κ_p_ and ρ_p_ which represent the compressibility and density of the fluid and particle, respectively, as well as on * Φ*($\tilde{\kappa}, \tilde{\rho}$), which is the acoustic contrast factor. Other parameters are the wave number (2π/λ) denoted by k, and the acoustic energy density, E_ac_. The acoustic radiation force also depends on z, i.e., the position of the particle along the wave propagation axis, and p_a_, the pressure amplitude, and c_0_, the speed of sound in the medium. **Supplementary Equation 2.** A particle in an acoustic standing wave field has a characteristic acoustophoretic mobility which is determined by the particle radius* α*, the viscosity of the medium* η*, and the acoustic contrast factor* Φ*. **Supplementary Equations 3.** Equations for the calculations of separation efficiencies (1, 2), whereby C denotes particle concentrations and V denotes the volumes of the respective center (c) and side (s) fractions. **Supplementary Table. Supplementary Table 1.** Primer Sequences for RT-PCR, primers were purchased from Life Technologies or TAG Copenhagen A/S (Copenhagen, Denmark). **Supplementary Figures. Supplementary Figure S1.** Schematic illustration of an acoustic pressure field (solid blue line) and the resulting acoustic radiation force (dashed red line) in a microchannel cross-section. Red arrows indicate the direction of F^rad^ for particles with a positive acoustic contrast factor Φ. **Supplementary Figure S2.** RT-PCR calibration curve to assess the sensitivity of NBC detection for the* PHOX2B*,* TH* and* DDC* genes used in the study. Cq values for PDX-1 cells, PBPCs and ratios of 1:100 to 1:1,000,000 PDX:PBPCs (n = 1, mean of three technical repeats).

## Data Availability

Data are available from the corresponding author on request.

## Abbreviations

CTC: Circulating tumor cell
DDC: DOPA decarboxylase
GAPDH: Glyceraldehyde-3-phosphate dehydrogenase
HSC: Hematopoietic stem cell
MRD: Minimal residual disease
PBPC: Peripheral blood progenitor cell
PDX: Patient-derived xenograft
PHOX2B: Paired-like homeobox 2B
TH: Tyrosine hydroxylase
