# Supplementary material for: Label-free separation of neuroblastoma patient-derived xenograft (PDX) cells from hematopoietic progenitor cell products by acoustophoresis
Source: Stem Cell Res Ther. 2021 Oct 15;12:542. doi: 10.1186/s13287-021-02612-2 (PMC8518319; doi:10.1186/s13287-021-02612-2)
Supplement: Supplementary file 1 — Additional file 1. Supplementary Equations. Supplementary Equation 1. In an ultrasonic standing wave field, the acoustic radiation force Fzrad acts on a particle that has a non-zero acoustic contrast factor, Φ. The magnitude of the force acting on the particle depends on the radius ɑ, κ0, ρ0, κp and ρp which represent the compressibility and density of the fluid and particle, respectively, as well as on Φ($\tilde{\kappa}, \tilde{\rho}$κ~,ρ~), which is the acoustic contrast factor. Other parameters are the wave number (2π/λ) denoted by k, and the acoustic energy density, Eac. The acoustic radiation force also depends on z, i.e., the position of the particle along the wave propagation axis, and pa, the pressure amplitude, and c0, the speed of sound in the medium. Supplementary Equation 2. A particle in an acoustic standing wave field has a characteristic acoustophoretic mobility which is determined by the particle radius α, the viscosity of the medium η, and the acoustic contrast factor Φ. Supplementary Equations 3. Equations for the calculations of separation efficiencies (1, 2), whereby C denotes particle concentrations and V denotes the volumes of the respective center (c) and side (s) fractions. Supplementary Table. Supplementary Table 1. Primer Sequences for RT-PCR, primers were purchased from Life Technologies or TAG Copenhagen A/S (Copenhagen, Denmark). Supplementary Figures. Supplementary Figure S1. Schematic illustration of an acoustic pressure field (solid blue line) and the resulting acoustic radiation force (dashed red line) in a microchannel cross-section. Red arrows indicate the direction of Frad for particles with a positive acoustic contrast factor Φ. Supplementary Figure S2. RT-PCR calibration curve to assess th [file 13287_2021_2612_MOESM1_ESM.docx]

**Supplementary Material**

**Label-free separation of neuroblastoma patient-derived xenograft (PDX) cells from hematopoietic progenitor cell products by acoustophoresis**

Franziska Olm^1^, Lena Panse^1, 2^, Josefina H. Dykes^3^, Daniel Bexell^4^, Thomas Laurell^5^, and Stefan Scheding*^1,6^

^1^ Lund Stem Cell Centre & Division of Molecular Hematology, Department of Laboratory Medicine, Lund University, Lund, Sweden

^2^ Department of Biotechnology, Technical University Berlin, Berlin, Germany

^3^ Division of Haematology and Transfusion Medicine, Department of Laboratory Medicine, University and Regional Laboratories, Lund, Sweden

^4^ Division of Translational Cancer Research, Department of Laboratory Medicine, Lund University Cancer Center, Lund University, Lund, Sweden

^5^ Division of Nanobiotechnology and Lab-on-a-chip, Department of Biomedical Engineering, Lund University, Lund, Sweden

^6^ Department of Haematology, Skåne University Hospital, Lund, Sweden

**Supplementary Equations**

$$F_{z}^{rad}= \frac{4}{3} \pi\phi\left( \tilde{\kappa},\tilde{\rho} \right)ka^{3}E_{ac}sin(2kz)$$

$$E_{ac}=\frac{p_{a}^{2}}{4\rho_{0}c_{0}^{2}}; \phi\left( \tilde{\kappa},\tilde{\rho} \right)= \frac{5\tilde{\rho}- 2}{2 \tilde{\rho}+ 1}- \tilde{\kappa} ; \tilde{\kappa} = \frac{\kappa_{p}}{\kappa_{0}} ; \tilde{\rho}= \frac{\rho_{p}}{\rho_{0}}$$

**Supplementary Equation 1.** In an ultrasonic standing wave field, the acoustic radiation force F_z_^rad^ acts on a particle that has a non-zero acoustic contrast factor, 𝜙. The magnitude of the force acting on the particle depends on the radius ɑ, κ_0_, ρ_0_, κ_p_ and ρ_p_ which represent the compressibility and density of the fluid and particle, respectively, as well as on 𝜙(𝜅̃, 𝜌̃), which is the acoustic contrast factor. Other parameters are the wave number (2π/λ) denoted by k, and the acoustic energy density, E_ac_. The acoustic radiation force also depends on z, *i.e.,* the position of the particle along the wave propagation axis, and p_a,_ the pressure amplitude, and c_0,_ the speed of sound in the medium.

$${Mobility}_{ac}= \frac{a^{2}\phi}{\eta}$$

**Supplementary Equation 2.** A particle in an acoustic standing wave field has a characteristic acoustophoretic mobility which is determined by the particle radius 𝑎, the viscosity of the medium 𝜂, and the acoustic contrast factor 𝜙.

${\% Relative recovery}_{center}=\frac{C_{C} \times V_{C}}{C_{C} \times V_{C} + C_{S} \times V_{S}} \times100 \%$ (1)

${\% Relative recovery}_{side}=\frac{C_{S} \times V_{S}}{C_{C}\times V_{C} + C_{S} \times V_{S}} \times100 \%$ (2)

**Supplementary Equations 3.** Equations for the calculations of separation efficiencies (1,2), whereby C denotes particle concentrations and V denotes the volumes of the respective center (c) and side (s) fractions.

**Supplementary Tables and Figures**

**Supplementary Table 1**: Primer Sequences for RT-PCR, primers were purchased from Life Technologies or TAG Copenhagen A/S (Copenhagen, Denmark).

| **Gene** | **Primer** | **Sequence 5´→ 3´** |
| --- | --- | --- |
| *GAPDH* | Forward | CACTCCACCTTTGACGC |
|  | Reverse | GGTCCAGGGGTCTTACTCC |
| *PHOX2B* | Forward | AACCCGATAAGGACCACTTTTG |
|  | Reverse | AGAGTTTGTAAGGAACTGCGG |
| *TH* | Forward | GGGCTGTGTAAGCAGAACG |
|  | Reverse | AAGGCCCGAATCTCAGGCT |
| *DDC* | Forward | TGAGTTTGAGTCACTGGTGC |
|  | Reverse | TCTTTGATGTGTTCCCAGGC |

**
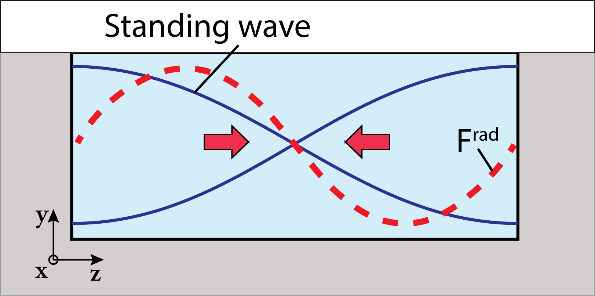
**

**Supplementary Figure S1:** Schematic illustration of an acoustic pressure field (solid blue line) and the resulting acoustic radiation force (dashed red line) in a microchannel cross-section. Red arrows indicate the direction of F^rad^ for particles with a positive acoustic contrast factor Φ.


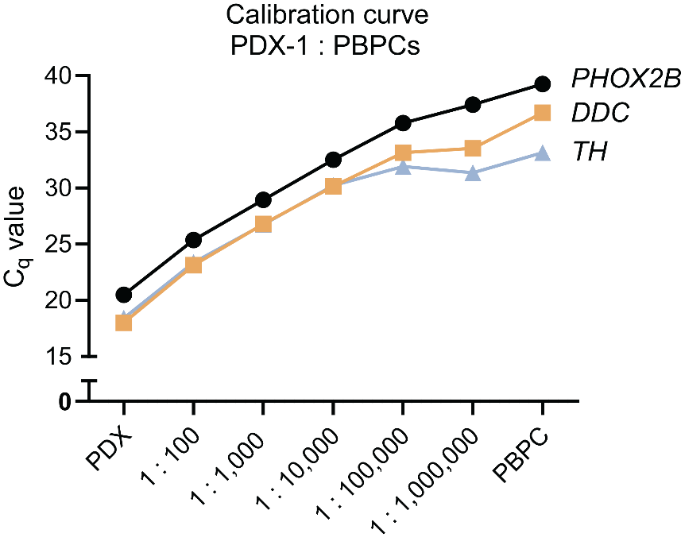


**Supplementary Figure S2:** RT-PCR calibration curve to assess the sensitivity of NBC detection for the *PHOX2B*, *TH* and *DDC* genes used in the study. C_q_ values for PDX-1 cells, PBPCs and ratios of 1:100 to 1:1,000,000 PDX:PBPCs (n=1, mean of three technical repeats).
